# A review of experimental models of focal cerebral ischemia focusing on the middle cerebral artery occlusion model

**DOI:** 10.12688/f1000research.51752.1

**Published:** 2021-03-26

**Authors:** Melissa Trotman-Lucas, Claire L. Gibson

**Affiliations:** 1 School of Psychology, University of Nottingham, Nottingham, NG7 2UH, UK

**Keywords:** Stroke, ischemia, focal ischemia, in vivo, refinement

## Abstract

Cerebral ischemic stroke is a leading cause of death and disability, but current pharmacological therapies are limited in their utility and effectiveness. *In vitro* and *in vivo* models of ischemic stroke have been developed which allow us to further elucidate the pathophysiological mechanisms of injury and investigate potential drug targets. *In vitro* models permit mechanistic investigation of the biochemical and molecular mechanisms of injury but are reductionist and do not mimic the complexity of clinical stroke. *In vivo* models of ischemic stroke directly replicate the reduction in blood flow and the resulting impact on nervous tissue. The most frequently used *in vivo* model of ischemic stroke is the intraluminal suture middle cerebral artery occlusion (iMCAO) model, which has been fundamental in revealing various aspects of stroke pathology. However, the iMCAO model produces lesion volumes with large standard deviations even though rigid surgical and data collection protocols are followed. There is a need to refine the MCAO model to reduce variability in the standard outcome measure of lesion volume. The typical approach to produce vessel occlusion is to induce an obstruction at the origin of the middle cerebral artery and reperfusion is reliant on the Circle of Willis (CoW). However, in rodents the CoW is anatomically highly variable which could account for variations in lesion volume. Thus, we developed a refined approach whereby reliance on the CoW for reperfusion was removed. This approach improved reperfusion to the ischemic hemisphere, reduced variability in lesion volume by 30%, and reduced group sizes required to determine an effective treatment response by almost 40%. This refinement involves a methodological adaptation of the original surgical approach which we have shared with the scientific community via publication of a visualised methods article and providing hands-on training to other experimental stroke researchers.

## Ischemic stroke disease

In the UK alone, over 100,000 strokes occur annually and approximately 1.2 million stroke survivors live with the consequences of a stroke. ^1^ Despite innovations in stroke research, treatment and rehabilitation, stroke remains the fourth leading cause of death within the UK. ^1^ Stroke is one of the commonest causes of complex disabilities in the UK, ^2^ with two thirds of patients leaving hospital with a post-stroke disability, costing the UK society an estimated £26 billion each year. ^3^ A person who suffers a stroke is highly likely to be affected by a variety of debilitating outcomes, including physical (swallowing, pain and sensory changes) and communication difficulties (speech, reading, writing and ability to understand) along with tiredness and fatigue, impacting on the quality of an individual's daily life. ^4^ A considerable proportion, ~85%, of strokes that occur are ischemic in nature ^5^ occurring when a cerebral vessel becomes blocked, preventing vital blood flow to the supply area of that vessel, ultimately leading to cellular damage and death.

The only current pharmacological treatment available with proven efficacy for ischemic stroke is thrombolysis treatment with recombinant tissue plasminogen activator (rtPA). However, a low number of patients qualify for rtPA treatment, due to strict eligibility criteria, combined with a narrow therapeutic time window of 4.5 hours results in only ~15% of patients receiving intra-venous rtPA treatment, a low acceptance value further exacerbated by a recanalization success rate of <50%. ^6, 7^ In addition to pharmacological treatment, mechanical clot removal via endovascular thrombectomy is increasingly used clinically for the treatment of large vessel occlusions, particularly with patients who respond poorly to rtPA treatment. ^6, 8^ This technique involves the removal of the clot blockage from directly within the affected vessel, allowing immediate reversal of the clot's impact on blood flow. The prompt restoration of vital blood flow to the ischemic area is the primary clinical goal, with the potential to salvage vital tissue and cellular function, thus reducing the spread of increasing ischemic damage. ^9^ This prompt recanalisation of blocked vessels is positively correlated with improved survival rates and improved recovery for ischemic stroke patients. ^10^

To allow for the development of useful clinical therapeutics for stroke treatment, the pathophysiology and mechanisms of disease/recovery need to be elucidated. For this to lead to constructive outcomes it is essential to utilise a combined approach of *in vitro* and *in vivo* models. Preclinical ischemic stroke models closely mimic the mechanisms of injury and subsequent recovery allowing investigation of potential clinically viable treatments. Although over 1,026 potential neuroprotective therapeutics have been tested preclinically, ^11^ breakthroughs have not passed beyond clinical trial often due to detrimental side effects, with current effective treatments concentrating on the chemical or physical unblocking of the occluded vessel. This lack of translation to the clinic of neuroprotective strategies continues, requiring us to re-examine the preclinical models used in research. Revisiting these models, to refine and evolve them to be more clinically representative and consistent between laboratories, aims to improve the robustness of preclinical studies and the reproducibility of data obtained.

## Preclinical models of ischemic stroke

### *In vitro and ex vivo* models of ischemic stroke

Due to the complex nature of ischemic stroke, it is not possible to successfully model using a single *in vitro* or *ex vivo* system. However, use of these bench-side systems allows the investigation of biochemical and molecular mechanisms involved during stroke-like ischemic conditions and to examine the resultant cellular damage. These systems can be utilised to determine pathways and cellular triggers involved in both necrotic and apoptotic cell death, ^12^ alongside the isolated investigation of cellular cascades that occur as a result of excitotoxicity. ^13^ Methods to induce stroke-like ischemic conditions *in vitro* may include chemical or enzymatic block of cellular metabolism. Metabolic inhibition is induced through application of chemicals such as 2-deoxyglucose, antimycin or sodium azide, ^14- 16^ which act to interact with the electron transport chain mimicking the energy depletion that occurs during cerebral stroke. Alternatively, a chemical-based model can use NMDA or glutamate receptor agonists to induce excitotoxic conditions via imitating the significant extracellular increase in glutamate that occurs during ischemia. ^17, 18^ Chemical or enzymatic bench-side models allow high-throughput testing, with ease of application and rapid responses, the method also allows isolated analysis of specific aspects of the molecular pathways involved. However, both approaches are reductionist in that they only attempt to mimic one aspect of the pathophysiological cascade and don't replicate the more complex interplay of mechanisms. A further disadvantage to the use of chemicals or enzymes to model ischemia is that these compounds may be difficult to wash out and therefore can disrupt the return to pre-insult conditions. The return to pre-insult conditions *in vitro* is undertaken to mimic the return of nutrients seen *in vivo* due to the return of blood flow to an area affected by stroke, known as reperfusion.

The most frequently used *in vitro* method to induce stroke-like ischemic conditions, is to remove all the available oxygen and glucose supply to the cells, known as oxygen-glucose deprivation (OGD). This is most often achieved by perfusing glucose-free media with a nitrogen/carbon dioxide mixture to displace oxygen, with subsequent experiments taking place inside a hypoxia chamber. Reperfusion conditions can be imitated through the reintroduction of glucose alongside a return to atmospheric oxygen. Induction of OGD leads to neuronal depolarisation within 10 minutes of onset, ^19^ with astrocytes showing immediate progressive depolarisation over the first 30 minutes of OGD. ^20^ OGD with reperfusion shows continued neuronal degradation over several hours following a return to 'normal' culture conditions, which combined with large extracellular glutamate increases ^21^ are both consistent with *in vivo* observations. ^22^ Experiments undertaken in hypoxic-only conditions are less representative of ischemic stroke but may better represent cerebral hypoxia conditions such as carbon monoxide poisoning. ^23, 24^ Currently, many *in vitro* ischemia models mimic global ischemia as they induce an insult to the overall brain slice or culture preparation and therefore do not mimic the clinical situation of a focal insult. Recently, Richard *et al.* have demonstrated the development of focal OGD in *ex vivo* cortical brain slices using targeted OGD media stream perfusion, perfusing the tissue surrounding the target area with artificial cerebrospinal fluid (aCSF) solution. They reported rapid neuronal depolarisation within the core OGD targeted area with slower progressive depolarisation in the surrounding aCSF perfused area, such as is seen in the penumbra. ^25^ The refinement of an *ex vivo* slice model of stroke to better represent the clinical presentation of cerebral ischemic events, may allow preclinical bench side investigations to occur in a more representative model.

To gain better relevance from bench side *in vitro/ex vivo* stroke models, the physiological micro-environment of the cells is critically important to gaining a true understanding of disease process and outcomes. The influence of oxygen, nutrients (including glucose), cell to cell contact and shear forces all need to be considered. With reference to the use of OGD as a key bench side ischemia-inducing model, oxygen levels should be considered when designing and interpreting data from these experiments. Physiological arterial blood oxygen concentrations differ significantly from external atmospheric levels. Maintenance at physiologically relevant oxygen levels has been shown to increase survival, cell proliferation and dopaminergic neuron differentiation in culture. ^26^ Whereas, high oxygen levels affect not only basal functioning but also response to challenge, potentially providing resistance to stroke related oxidative stressors. ^27^ In addition to high oxygen levels used within *in vitro* culture systems, glucose is also poorly matched to physiological concentrations. Cell culture glucose concentrations are often up to 8x higher than those reported within the brain. ^12, 28, 29^ Glucose is used in culture maintenance at concentrations proposed to be present within the brain during severe hyperglycaemia and shown to affect neuronal viability. ^28^ A rethink of the use of long-standing outdated culture media maintenance and methods is required to improve physiological relevance.

One step towards improving physiological relevance of *in vitro* stroke models is the introduction and use of co-culture models which are based on multiple cell types, typically placed in repeated monolayers, allowing substrates and signalling molecules to pass between several cell types. This approach is common for modelling the blood brain barrier and has improved our understanding of neurovascular changes that occur during ischemia. Developments in 3D cell culture have the potential to improve the physiological relevance of bench side ischemia models even further. Cells cultured in 3D, on either scaffolds or a scaffold-free system, show natural cell shapes, prevalent cell to cell junctions, well differentiated cell types and a greater response to mechanical stimuli, overall improving physiological relevance compared to 2D culture models. ^12, 30^ Cells show lower mortality rates, resistance to nutrient deprivation and drug insults, this is alongside the presence of a gradient based availability of culture media nutrients more like that of a physiological state. Popularity and interest in the use of 3D cultures for *in vitro* modelling is increasing and they offer an additional way to investigate potential neuroprotective treatments prior to the need for *in vivo* approaches, reducing ethical impact. In addition to the use of bench side models of multiple cell types to increase physiological relevance, the use of microfluidic devices is also being explored to improve the relevance and applicability of bench side disease models, often termed organ-on-a-chip models. ^31^ Microfluidic devices, since the advent of lithography in 2001, ^32^ use a micro-engineered culture platform that can be utilised to mimic blood flow in health and disease models, alongside allowing the isolated investigation of upstream and downstream signalling within and between cells, due to microchannels that allow axonal growth along them. Since the early 2000s there have been multiple developments in the field of biomicrofluidics to try to overcome some of the limiting factors of this type of cell culture, as this is still constrained to the typical 2D rigid culture techniques discussed earlier. Improvements towards a brain-on-a-chip model are still required, but the use of microfluidics devices for axon-specific responses, cell-cell interaction and high-throughput screening are of great interest to the preclinical stroke research community, and with advancements in the field may prove to be another key step in the drug translation process that will help improve the positive potential of tested neuroprotective therapeutics within the clinic. Although *in vitro* research provides a platform to determine and understand cell-specific responses to stroke-like conditions the complexities of clinical stroke often require these methods to be combined with *in vivo* approaches.

### *In vivo* models of ischemic stroke

The significant lack of neuroprotective therapeutics in the clinic has led to extensive work aimed at improving the reproducibility of preclinical stroke models and to ensure they reflect as closely as possible the clinical disease. ^33- 36^ To date, multiple preclinical stroke models have been developed to reproduce both global and focal ischemic stroke. Whereas global ischemia models more closely mimics the situation of cardiac arrest, focal ischemia models represent the typical clinical presentation of ischemic stroke. Various models are available for use in a variety of animal species, however the use of rodents, specifically rats and mice, is the most common. This is likely to be due to low acquisition and husbandry costs associated with these species, combined with simple effective monitoring methods and ease of tissue processing. ^24^ To try and address the gap in clinical translation for stroke treatments the Stroke Therapy Academic Industry Roundtable (STAIR) published a number of recommendations including the need for potential therapeutics to be assessed across a number of species, initially rodents followed by studies using gyrencephalic species. ^33, 37^ The use of higher-order species is necessary to overcome the evolutionary differences between rodents and primates such as the corticospinal tract descending from the motor cortex. ^38^ Larger species may also be suitable for investigations utilising endovascular techniques, such as clot retrieval and stenting, techniques used more frequently in the clinic. However, due to cost, availability and ethical considerations with larger higher-order species, rodents still play a key role in research to ensure experimental power in addition to testing the safety and efficacy of potential neuroprotective treatments.

The most frequently used *in vivo* model of focal ischemia is intraluminal suture middle cerebral artery occlusion (iMCAO), ^39^ which has enhanced our knowledge of the pathophysiology of cerebral ischemia including penumbra development and functioning, blood brain barrier injury, cell death pathways and inflammatory processes related to cerebral ischemia. Developed initially for use in the rat, ^40^ in which infarction success rates are 88-100%, ^41^ the method was later modified by Longa *et al.* ^42^ and subsequently adapted for use in mice ^43^ where the model is increasingly being used particularly due to the availability of transgenic mouse strains. Induction of iMCAO is achieved by the insertion of a flexible monofilament into either the common carotid artery (CCA) ^40^ or external carotid artery (ECA). ^42^ Access into the CCA requires the vessel to be ligated for incision. ^40^ Similarly, for filament access, the ECA is typically transected in the modified iMCAO method. ^42^ Once in the vessel lumen, the filament is advanced into the internal carotid artery (ICA) and to the bifurcation of the middle cerebral artery (MCA). Once *in situ*, at the origin of the MCA, the filament head impedes blood flow into the MCA territory and is left *in situ* for the duration of ischemia. The utility of this model allows for accurate control of occlusion duration, allowing for either permanent ischemia where the filament remains in place, or transient ischemia where the filament is removed to allow reperfusion of blood flow to the MCA territory. Utilising the iMCAO model, post-stroke effects can be examined months following the initial insult. Vessel access is obtained without craniectomy and therefore avoids cranial damage associated with craniectomy which could impact on intracranial pressure changes and post-stroke outcomes such as reduced lesion volume. ^44, 45^ Use of iMCAO results in large infarct volumes encompassing both the striatum and cortex, ^46, 47^ however, longer durations of iMCAO can lead to hypothalamic damage which occurs rarely in humans and can lead to hyperthermic responses in rats and poor temperature control in mice. ^48, 49^ Following iMCAO, rodents can experience a range of side-effects that negatively impact the welfare of animals, including but not limited to, significant weight loss, abnormal or reduced motility, difficulty eating/drinking and mortality. These outcomes can be moderated through enhanced pre and post-surgery care; detailed recommendations to support this have been highlighted by the IMPROVE guidelines. ^50^

The filament selection for use within iMCAO also plays a key role within the model, as unsuitable filament selection can lead to inadequate occlusion and filament-induced secondary subarachnoid haemorrhage due to arterial rupture. ^51^ This has led to an increase in the use of standardised silicone-coated filaments to attempt to improve reproducibility and the use of laser doppler flowmetry to confirm correct filament placement and monitor occlusion duration. ^52^ Although the iMCAO model is not appropriate to study the effect of thrombolysis treatment in conjunction with tested therapies it does recapitulate occlusion of the MCA in the clinic, which is the most common location of thromboembolic stroke in humans. ^53^ However, placement of the intraluminal filament into the origin of the MCA is an all or nothing approach, which does not reflect the clinic where human stroke is frequently not caused by a complete occlusion, additionally the model does not replicate the event of partial spontaneous reperfusion that can occur in patients within 48 hours of stroke onset. ^54, 55^ Furthermore, the model does not profile the slow clot disruption that occurs following rtPA administration, instead showing surge reperfusion upon filament removal. ^56^ More recently however, the model has become increasingly relevant due to the advent of interventional mechanical thrombectomy in the clinic. In 2015, five randomised controlled clinical trials reported beneficial effects of endovascular intervention therapy in treating patients with large vessel occlusions, with or without rtPA treatment. ^57^ This beneficial effect was correlated with the abrupt recanalisation of the vessel and rapid reperfusion of the ischemic zone, corresponding to the mechanisms of the iMCAO model. The positive outcome of endovascular thrombectomy, as reported in the clinical trials, suggests this mechanical treatment will become the primary therapy for large vessel occlusion in the clinic; ^56^ renewing relevance of the established iMCAO model as a model of endovascular thrombectomy.

In addition to iMCAO, focal ischemia can also be induced *in vivo* by direct occlusion of the vessel of interest, using a cranial window to either clamp, ligate or cauterise the vessel in situ. The most used cranial entry focal ischemia method was developed in 1981 by Tamura *et al.*, using distal MCA occlusion, similar to the occlusion location obtained using iMCAO, inducing combined cortical and striatal lesions. ^58^ Like the iMCAO method, craniotomy models can be used to induce either permanent or transient focal ischemia - although relying heavily on the experimenter's ability to reduce potential localised cerebral damage for both scenarios. Similarly, the return to perfusion following transient ischemia using this method results in a sudden prompt mechanical perfusion, again unlike the presentation of reperfusion in the clinic. Furthermore, access to the vessel of interest through the skull has been shown to induce cortical spreading depressions and inflammatory responses. ^59^

Additional stroke models are available that utilise specialised mechanisms to induce ischemia, typically in rodents, including thromboembolic, endothelin-induced and photochemical models. Embolic models can be broadly categorised into thromboembolic and non-clot embolic models; the former utilising the induction of localised clots or the insertion of spontaneous/thrombin-induced clots and the latter utilising non-clot methods such as micro/macrospheres to occlude vessels. ^52, 60, 61^ Since most human strokes are caused by thromboembolism, thromboembolic stroke models may more closely represent the clinical disease pathology. However, there is a lack of a single standardized embolic stroke model leading to multiple occlusion induction methods, a further method that has shown increasing use is the localised injection of thrombin directly into a craniotomy exposed MCA bifurcation. ^62^ A significant advantage of this thromboembolic technique is the opportunity to test thrombolysis treatments alone or in combination with neuroprotective agents. ^63, 64^ Additionally, the use of rtPA to lyse the clot in thromboembolic models results in a reperfusion profile that is closer to the reperfusion profiles of rtPA treated patients, contrasting the sharp reperfusion profile following filament removal using iMCAO, increasing the relevance of clot emboli models. ^56, 65^ However, infarct location and volume can be highly variable using thromboembolic stroke models along with low seven-day survival rates, ^66^ which impacts the reproducibility of thromboembolic stroke models and their utility for longitudinal studies. In addition to the difficulties experienced in controlling lesion location, the duration of the ischemic insult is difficult to control in these models with some animals showing spontaneous recanalisation, ^62, 67, 68^ a further disadvantage to the model is the potential for spontaneous clot formation following embolism disruption. ^69^ The severity of this preclinical stroke model also has a high mortality rate of >30%, typically within the first 24 hours preventing longitudinal study. ^50, 70^ Non-clot embolic methods can include the injection of silicone or collagen into the ICA of rodents ^71, 72^ or, for example, the injection of micro/macrospheres resulting in microembolisations causing multifocal and heterogenous lesions. ^73, 74^ Administration of micro/microspheres results in an immediate reduction in cerebral blood flow (CBF) that becomes progressively stronger over the first 3-12 hours following sphere injection, this in turn leads to a slow infarct development, suggesting a longer therapeutic time window than other stroke models for instance iMCAO. ^74^

Endothelin models of ischemia employ the peptide Endothelin-1, a long-lasting potent vasoconstrictor, to induce vessel occlusions. ^75, 76^ The peptide is typically applied using stereotaxic injection methods ^77, 78^ or craniotomy ^79, 80^ applied directly onto an exposed cerebral vessel causing a constriction of the vessel reducing CBF to the vessel territory for up to 22 hours post-injection ^81^ followed by gradual reperfusion. The severity and duration of an insult can be adjusted according to the concentration of the peptide at application. The sustained reduction in CBF and subsequent gradual reperfusion profile, alongside gradual lesion development resembles the evolution of clinical stroke. The topical application method of the peptide is a source of variability in this model, due to the difficulty in ensuring consistent diffusion. To reduce this variability intracortical injection of the peptide to sensorimotor areas has been developed. ^82, 83^ With this injection based method, care must be taken to avoid the compound entering the ventricles to avoid significant negative welfare outcomes such as barrel rolling or seizures. ^69^ Another targeted approach of MCA occlusion induction is the photothrombotic stroke model, which induces localised permanent infarcts of the cortex by introducing a photosensitive dye (e.g. Rose Bengal) into the cardiovascular system and illuminating this through the skull using a specific wavelength of light. Targeting small vessels, this illumination activates the photosensitive dye resulting in endothelial damage due to oxygen species formation, causing platelet activation and aggregation. ^84, 85^ These together result in the formation of an occlusion and consequently rapid ischemia to the vessel territory, leading to the development of cortical lesion. ^86^ This method produces reproducible and localised cortical lesions, with minimal variation in lesion volume, ^86^ the small cortical lesions and low mortality associated with the model lend well to longitudinal study however, a lack of penumbral tissue within the lesion reduces the translational impact of the model, as the penumbra is the target tissue of interest for neuroprotective strategies. Photothrombotic induction requires the use of light sources, these can act as a heat source. The illumination can have a heating effect on the skull and subsequently the brain, therefore it is important that care over temperature must be taken and where possible cold light sources used, with exposure and distance from the skull/brain considered. ^50, 82^ Furthermore, due to the systemic nature of the dye the model is unsuitable for preclinical drug studies.

## Refining the *in vivo* model of iMCAO

The lack of lab to clinic translation of neuroprotective therapies is an ongoing issue and understanding the reasons for this is key to improving the future clinical potential of preclinical stroke research. Many in the field have deliberated the difference in outcomes between preclinical data and the clinical trial outcomes of the many tested neuroprotective agents. ^35, 87- 90^ Reasons conferred for the disparity include; variations in treatment implementation timepoints, queries about dose effectiveness between species, outcome measure disparities between preclinical (typically lesion volume) and clinical (mainly death rate and disability) also, whether *in vivo* models effectively model drug efficacy. ^89, 90^ Moreover, systematic meta-analyses suggest the introduction of bias into data as a result of poor study design and subsequent study implementation. ^90- 92^ With sources of bias coming from both internal (e.g. selection or, performance or attrition bias, small sample sizes and low overall power) and external (e.g. publication bias and the use of exclusion criteria such as co-morbidities, age and sex) factors, acting to weaken the strength of studies.

The most commonly used *in vivo* model of ischemic stroke, iMCAO, has been shown to produce lesion volumes with large standard deviations even though rigid surgical and data collection protocols are followed, ^88, 93, 94^ with 40% standard deviation accepted as reproducible infarct outcomes. ^95^ The iMCAO model relies heavily on collateral flow through the Circle of Willis (CoW), a network of vessels connecting vertebral and carotid circulation, particularly at reperfusion due to ipsilateral CCA ligation. Reliance on the CoW may afford some of the variability in lesion volume reported following iMCAO, due to anatomical variations in its structure, particularly within C57BL/6 (B6) mice commonly used in preclinical stroke studies. CoW variation has been shown to occur in B6 mice, with 90% showing one or both posterior communicating arteries (PcomA) missing. ^47^ Kitagawa *et al.*, reported that the patency of the PcomA is a significant determinant of ischemic damage area following iMCAO. ^96^ Furthermore, additional models of cerebral ischemia such as the bilateral common carotid artery occlusion model also report variations attributed to PcomA patencies, in both lesion volume and CBF during ischemic events, when compared across mouse strains. ^97- 99^ Variation in experimental design and husbandry techniques may also impact the potential translational ability of iMCAO studies. Recommendations provided by the IMPROVE guidelines ^50^ on experimental design, husbandry, enrichment, analgesia and post-operative care may go some way to improving standardisation and welfare across iMCAO studies, reducing variability not only within but between studies. This may contribute towards improving the reproducibility of preclinical stroke research, allowing the focus to be on the experimental question and assessing this across research groups with a collaborative approach to research. The IMPROVE guidelines also stress the need for comprehensive reporting of confounding factors in published research, to allow correct interpretation of presented data, improving the reliability and adding value to the information provided, not only improving welfare but also consistency across the stroke research field.

We have previously reported an alternative surgical approach to iMCAO that improves reperfusion to the ischemic hemisphere, reduces lesion volume variability and subsequently reduces group sizes estimated, using power calculation, to determine an effective treatment response in terms of lesion volume. ^100, 101^ Typically, in iMCAO, entry to the cerebrovascular system is obtained via an incision into the CCA or transection of the ECA. However ECA transection, in rats, has been shown to induce ischemic lesions within the muscles controlling mastication and swallowing, ^102^ resulting in changes to drinking behaviour and weight-loss post-MCAO. ^103^ Preclinical stroke models, including iMCAO, that utilise ECA transection do so to minimise interruption to circulatory flow, in an attempt to maintain anatomic integrity to improve post-stroke reperfusion. ^104^ We reported that avoidance of ECA ligation, alongside the introduction of analgesia, appeared to reduce weight loss, without impacting lesion volume when using the modified iMCAO method. ^101^ Although limitations in experimental design did not allow the elucidation of the direct effects of analgesia versus ECA ligation avoidance, systematic administration of an analgesia regime across all experimental groups prevented this becoming a confounding variable. Analgesia use should be utilised in preclinical stroke research as a means of good practice, particularly as many preclinical stroke models involve surgical interventions, with the type of analgesia carefully selected in relation to any potential interference with a study's scientific outputs. The IMPROVE guidelines provide a comprehensive discussion on the use of analgesics within preclinical stroke research, providing recommendations to promote analgesia use within the field. ^50^

The alternative iMCAO method we established improves mouse well-being and removes reliance on the CoW for collateral flow during reperfusion, importantly reducing lesion volume variability. ^100, 101, 105^ As lesion volume is often the primary outcome measure for neuroprotective *in vivo* stroke research, large variations within this data can result in low statistical power if sample sizes are not accordingly adjusted to account for this variability. ^93^ Typically, preclinical stroke studies have low statistical power, a statement supported recently by a meta-analysis revealing that on average, studies show 59% power to detect a 30% inter-group difference. ^93^ The low power of experimental stroke studies, in simple terms, could be improved by increasing group sizes, however, this is contrary to the research community's drive to implement the 3Rs principles, where it is important to ensure studies are correctly powered. ^106, 107^ The need to use appropriate animal numbers in experimental research, in line with ethical requirements and the 3Rs principles is leading researchers to re-address the models used and experimental designs undertaken, in addition to the need to overcome the translation roadblock in neuroprotective stroke research. The iMCAO model refinement is one step within the preclinical stroke community towards reducing animal numbers. This is due to reduced variability in outcome data, improving statistical power, and leading to reduced animal numbers required per experimental group to determine treatment effect. We demonstrated that undergoing CCA repair following iMCAO increased perfusion to the ipsilateral hemisphere compared to a typical CCA ligation iMCAO method ^100^ and that use of the CCA repair model could require group sizes 39% smaller than with use of the traditional CCA ligation technique, to attain 80% power with a significance level of 0.05 and an anticipated 30% difference in lesion volume between groups. ^101^ For a typical year, in this instance 2019, a Pubmed search using the search term '((MICE) OR (mouse)) and (MCAO))' determined there were 210 original research articles reporting data from mice undergoing iMCAO. Based on data we reported previously, ^101^ a group size of 58 animals per group (control and experimental condition) per article can be assumed - this group size would result in 24,360 animals per year, but of course that does not include excluded animals alongside those animal experiments that were either not included in published studies or were never completed and analysed for publication. A 39% reduction in group size, as reported following use of the CCA repair iMCAO method, ^101^ would reduce the number of animals required across all those studies published in 2019 by 9500.

## Scientific applications of refinements

The iMCAO CCA repair method we have highlighted here could have impact on other vascular surgical models within the preclinical stroke research field and potentially beyond. For example, embolic stroke models that deposit a clot into the cerebral vascular system via a carotid artery could be adapted to incorporate the demonstrated CCA repair method. Particularly, by accessing the vessel lumen through the CCA rather than the ECA, alongside the vessel sealing aspect of the refinement allowing bilateral CCA perfusion to the affected ischemic area. The refined iMCAO model using the CCA repair technique may also be of use in additional *in vivo* research applications, for instance therapeutic drug delivery in experimental models or intra-arterial delivery (IA) of stem cells. Currently there have been over 50 IA cell delivery studies within the stroke field ^108^ with potential IA applications in other research fields. Argibay *et al*. 2017, reported that maintaining flow through the CCA resulted in uniform cerebral cell distribution following IA cell delivery, with engraftment of labelled mesenchymal stem cells (MSC) visualised using T2-weighted magnetic resonance imaging. ^109^ The group utilised Longa's ^42^ transient iMCAO method in rats, with both filament insertion and MSC injection via a transected ECA, with the CCA temporarily tied for the duration of iMCAO. ^109^ CCA repair, reported previously to be successful in rats, ^110, 111^ alongside our recent method development in mice, ^101, 105^ would negate the requirement to transect the ECA, therefore, removing the negative welfare outcomes reported following ECA transection. ^102, 103, 110^ Although many IA stem cell delivery experimental models utilise rats, there are examples of these studies conducted in mice. Ge *et al*. 2014, utilised transgenic mice expressing green fluorescent protein to assess the impact of MSC size on neuronal health in naive animals undergoing IA MSC infusion, during which multiple arteries were transected and coagulated to enable catheter placement. ^112^ The delicate nature and small size of mouse arteries, compared to the typically more robust nature of rat arteries, increases the difficulty and applicability of IA procedures, potentially discouraging the development of improved methods utilising mice, even though the use of mice over rats opens-up the possibility of transgenic line use, and the targeted downstream investigations the use of transgenic animals allows. Use of the CCA repair technique provides an attractive solution to the problem of vessel sealing following arterial incision in mice, removing reliance on the CoW for collateral flow across the brain. Studies using IA delivery of MSC alongside stroke induction, typically induce iMCAO via the Longa *et al*. ^42^ method of ECA transection for filament introduction. This is due to the need to maintain CCA flow for microneedle injection of cells, as evidence suggests the injection of MSCs into a no flow vessel results in micro stroke lesions due to the preserved flow within the injection vessel. It is therefore possible that the repair technique could have an impact in this field, allowing the induction of stroke conditions but allowing CCA perfusion post iMCAO induction to allow IA microneedle injection. For catheter-based IA models, the sealing of the CCA vessel rather than the ligation of this vessel may be able to be undertaken with adaption of the CCA repair technique.

## Dissemination and uptake of the refined *in vivo* model of iMCAO

Our overall aim in producing a refined model of iMCAO was to achieve 3Rs impact within the field of experimental stroke. The importance of this work is in demonstrating that reduced variability in animal stroke studies has the potential to increase animal wellbeing, reduce the number of animals used and potentially increase the efficacy of animal studies in detecting treatment effects. Within a scientific field, results are typically disseminated through scientific publications and any methodological adaptations, if successful, tend to be taken up relatively slowly by the community. Researchers prefer to use 'established techniques' and are resistant to changing methods they have successfully applied for many years. However, there is a real commitment within the stroke community to improve the reliability and reproducibility of animal stroke models so that they may better inform the design and outcomes of clinical trials, evidenced by recent steering papers such as the IMPROVE guidelines. ^113^ We have engaged proactively with the stroke community to deliver hands-on training to numerous labs in the UK, Germany and USA involving over 20 researchers. To extend the reach into the community we published a visualised method of our refined approach ^105^ which has been viewed over 8000 times since publication in early 2019 and over 2000 times in the last six months. Subsequent work published by ourselves and others brings attention to this refined approach and we continue to offer it as our standard training model to incoming members of our group and in other research groups who contact us for training advice. ^50^ We feel that by engaging with some of the key preclinical stroke labs worldwide we will speed up the adoption of this refined technique.

## Conclusion

To further understanding of stroke pathophysiology and to develop novel clinical therapeutics experimental models of ischemic stroke are beneficial. Such models, based on both *in vitro* and *in vivo* approaches, have greatly enhanced our understanding of stroke physiology and pathology. However, there is still a lack of successful clinical therapies being translated from preclinical bench work to clinical use. As various types of *in vitro* and *in vivo* models have been developed selection of the most appropriate model to test the therapeutic is key. However, it is also necessary to constantly strive to improve the validity of the models being used in terms of their relevance to the clinical situation and to improve animal wellbeing. Here, we focus on the middle cerebral artery occlusion model which is the most commonly used *in vivo* stroke model. The refined surgical approach described here reduces the variability, in lesion volume, associated with this model and improves the welfare of experimental animals.

## Data availability

No data are associated with this article.
