# Supplementary material for: A review of experimental models of focal cerebral ischemia focusing on the middle cerebral artery occlusion model
Source: F1000Res. 2021 Jun 11;10:242. Originally published 2021 Mar 26. [Version 2] doi: 10.12688/f1000research.51752.2 (PMC8127011; doi:10.12688/f1000research.51752.2)
Supplement: Peer review history

# Peer review: Reviewer response for version 1

**DOI:** 10.5256/f1000research.54949.r82456

**Published:** 2021-05-12

**Authors:** Ruoli Chen

**Affiliations:** 1 School of Pharmacy and Bioengineering, Keele University, Staffordshire, UK

This is an excellent review article summarising experimental models of ischaemic stroke. It not only provides introduction with critical comments on both in vitro and in vivo preclinical models of ischaemic stroke, but also puts forward some proposals to refine iMCAO, the most commonly used in vivo model of ischameic stroke. iMACO produces lesion volumes with large standard deviation, thus has low statistical power. The authors have previously reported an alterative surgical approach to iMCAO, e.g, avoidance ECA ligation, providing analgesia. In this review article, the authors highlight iMCAO CCA repair method to further reduce lesion volume variability.

Is the review written in accessible language?

Yes

Are all factual statements correct and adequately supported by citations?

Yes

Are the conclusions drawn appropriate in the context of the current research literature?

Yes

Is the topic of the review discussed comprehensively in the context of the current literature?

Yes

Reviewer Expertise:

neuroprotection, blood brain barrier, hypoxia

I confirm that I have read this submission and believe that I have an appropriate level of expertise to confirm that it is of an acceptable scientific standard.

# Peer review: Reviewer response for version 1

**DOI:** 10.5256/f1000research.54949.r82453

**Published:** 2021-04-26

**Authors:** Lorraine M. Work

**Affiliations:** 1 Institute of Cardiovascular & Medical Sciences, University of Glasgow, Glasgow, UK

The review by Trotman-Lucas & Gibson is comprehensive and covers the various experimental models of ischaemic stroke available having also described the in vitro models which may be used to support in vivo studies. The detail is appropriate for a review, it is well written and supported through citation of the primary research material. 

 One suggested addition to improve the ease of access to the information described and discussed would be the inclusion of a summary table highlighting key considerations of each model (e.g. whether reperfusion can be achieved, ease of surgical technique required, limitations).

Is the review written in accessible language?

Yes

Are all factual statements correct and adequately supported by citations?

Yes

Are the conclusions drawn appropriate in the context of the current research literature?

Yes

Is the topic of the review discussed comprehensively in the context of the current literature?

Yes

Reviewer Expertise:

Preclinical models of experimental stroke

I confirm that I have read this submission and believe that I have an appropriate level of expertise to confirm that it is of an acceptable scientific standard.

# Response

**Published:** 2021-05-20

**Authors:** claire gibson

**Affiliations:** university of nottingham, UK

Thank you for these thoughtful comments from the reviewer.  We agree that a table would be informative and have now included this in our revision.
